# Effect of genetic distances of different genotypes of maize on the authenticity of single seeds detected by NIR spectroscopy

**DOI:** 10.3389/fpls.2024.1361328

**Published:** 2024-02-29

**Authors:** Yongqin Yang, Rashaun Candace Harrison, Dun Zhang, Binghui Shen, Yanlu Yan, Dingming Kang

**Affiliations:** ^1^ Ministry of Education of the People's Republic of China (MOE) Key Laboratory of Crop Heterosis and Utilization, College of Agronomy and Biotechnology, China Agricultural University, Beijing, China; ^2^ Department of Applied Physics, College of Science, China Agricultural University, Beijing, China; ^3^ Department of Electrical Engineering, College of Information and Electrical Engineering, China Agricultural University, Beijing, China

**Keywords:** NIR spectroscopy, maize, seed, authenticity identification, genetic distance

## Abstract

**Introduction:**

NIR spectroscopy combined with chemometric algorithms has been widely used for seed authenticity detection. However, the study of seed genetic distance, an internal feature that affects the discriminative performance of classification models, has rarely been reported.

**Methods:**

Therefore, maize seed samples of different genotypes were selected to investigate the effect of genetic distance on the authenticity of single seeds detected by NIR spectroscopy. Firstly, the Support vector machine (SVM) model was established using spectral information combined with a preprocessing algorithm, and then the DNA of the samples was extracted to study the correlation between genetic and relative spectral distances, the model identification performance, and finally to compare the similarities and differences between the results of genetic clustering and relative spectral clustering.

**Results:**

The results were as follows: the average accuracy of the models was 93.6% (inbred lines) and 93.7% (hybrids), respectively; Genetic distance and correlation spectral distance exhibited positive correlation significantly (inbred lines: r=0.177, *p*<0.05; hybrids: r=0.238, p<0.05), likewise genetic distance and model accuracy also showed positive correlation (inbred lines: r=0.611, p<0.01; hybrids: r=0.6158, *p*<0.01); Genetic clustering and spectral clustering results were essentially uniform for 94.3% (inbred lines) and 93.9% (hybrids), respectively.

**Discussion:**

This study reveals the relationship between the genetic and relative spectral distances of seeds and the accuracy of the model, which provides theoretical basis for the establishment of the standardized system for detecting the authenticity of seeds by NIR spectroscopic techniques.

## 1 Introduction

Since its domestication by humans 9,000 years ago, maize has played an increasingly diverse role in global agricultural systems and is an important food and feed crop in the world (Kennett et al., 2020). The purity and authenticity of maize hybrids is not only indication of seed identity, but also at least one of the most important indicators of seed quality. Related studies have shown that for every 1% reduction in seed purity, the yield of maize is reduced by 180 kg/ha (Zhao and Wang, 2013). Authenticity of maize inbred lines, on the other hand, is the critical factor in determining the authenticity of progeny hybrids (Frascaroli et al., 2007).

Currently popular detection methods, seed or seedling morphology testing is simple to operate, but the scope of application is narrow; field planting identification results are intuitive but the cycle time is long; protein fingerprinting technology experimental results are unstable; DNA fingerprinting technology is authoritative and accurate because of its detection of DNA molecular level differences; however, like protein fingerprinting technology, the method must be damaged samples, and requires a certain sample size, the required equipment, personnel costs and technology costs are higher, in addition to the testing process of the waste will also pollute the environment (Ertiro et al., 2015). Therefore, it is necessary to explore a more rapid, convenient, reliable and accurate technique for seed sample authenticity detection (ElMasry et al., 2019).

Near-infrared (NIR) spectroscopy is the qualitative and quantitative detection of hydrogen-containing groups (e.g. C-H, N-H, O-H, etc.) by analyzing the information of octave and combined frequency between them, which is widely used in the identification of the purity of crop seed varieties due to the advantages of non-destructive, rapid and high throughput (Osborne and Fearn, 1986). The PLS-DA classification model was able to achieve 80-100% accuracy in studies applying NIR spectroscopy to differentiate wheat seeds of different ploidy (Ziegler et al., 2016). In addition, previous studies have used chemometric algorithms such as multiple scattering correction (MSC), principal component analysis (PCA), and K-nearest neighbor algorithm (KNN) to test the authenticity of 520 rice samples of different quality grades and origins, and the classification results exceeded 90% (Teye et al., 2019). However, the former researchers mainly focused on factors including basic statistics, selection of classification methods, and data processing methods, while the effect of genetic differences between different samples, i.e. genetic distances, on the identification performance of the model has rarely been published, furthermore, there is as yet no systematic study on the correlation between genetic distances and relative spectral distances, and between the genetic clustering results and the spectral clustering results.

Therefore, in this experiment, different maize inbred lines and hybrids were selected to study the relationship between genetic distance and spectral distance among samples and the performance of the model discrimination. Specifically, the experiments included: 1) using spectral data of samples to build a SVM discrimination model combined with preprocessing; 2) using near-infrared spectral data to calculate the relative spectral distance among samples and carry out spectral clustering; 3) extracting DNA from samples, calculating genetic distance, and performing genetic clustering; 4) comparing and analyzing the results of the two clustering methods, as well as the relationships between relative spectral distance, genetic distance, and model discrimination performance among samples.

## 2 Materials and methods

### 2.1 Preparation of seed samples

In this research, 35 inbred lines and 33 hybrids were selected from the production base of maize seed in Zhangye City, Gansu Province, which were harvested in 2018 (**Table 1**). All the genotypes were the main inbred lines and hybrids in different maize planting areas in China. There were 150 grains of each genotype, totalling 10200 samples, were chosen from seeds with full kernels and free of pests and diseases. After moisture equilibration, the moisture content of all samples was maintained at 10% to 11%.

**Table 1:** Number, name and abbreviation of 68 maize seed samples.

No.	Variety name	Abbreviation	No.	Variety name	Abbreviation
1	PA 540	INBRED LINE 2-PA540	36	Jinbei 518	HYBRID 2-JB518
2	PA 21	INBRED LINE 4-PA21	37	Longyuan 3	HYBRID 3-LY3H
3	K2934	INBRED LINE 5-K2934	38	Dongdan 7512	HYBRID 4-DD7512
4	MB4	INBRED LINE 7-MB4	39	Zhengda 21	HYBRID 5-ZD12
5	Zheng 58	INBRED LINE 10-ZHENG58	40	Wuke 606	HYBRID 7-WK606
6	Chang 7-2	INBRED LINE 12-C7-2	41	Huanong 866	HYBRID 9-HN866
7	Jing 92	INBRED LINE 13-J92	42	Jindan 60	HYBRID 10-JD60
8	Jing 724	INBRED LINE 14-J724	43	Kehe 24	HYBRID 11-KH24
9	PH4CV	INBRED LINE 15-PH4CV	44	Kexing 216	HYBRID 14-KX216
10	PH6WC	INBRED LINE 16-PH6WC	45	Shangyu 3899	HYBRID 15-SY3899
11	351-14-35	INBRED LINE 22-351-14-35	46	Longdan 339	HYBRID 16-LD339
12	352	INBRED LINE 23-352	47	Ganxin 217	HYBRID 17-GX217
13	353	INBRED LINE 24-353	48	Longdan 10	HYBRID 19-LD10H
14	354	INBRED LINE 25-354	49	Longdan 8	HYBRID 20-LD8H
15	161	INBRED LINE 26-161	50	Ganyu 23	HYBRID 21-GY23
16	1520	INBRED LINE 27-1520	51	Xiongyu 582	HYBRID 22-XY582
17	1895	INBRED LINE 28-1895	52	Hongyu 601	HYBRID 23-HY601
18	4097	INBRED LINE 29-4097	53	Longdan 9	HYBRID 24-LD9H
19	4158	INBRED LINE 30-4158	54	Zhongdi 175	HYBRID 25-ZD175
20	4428	INBRED LINE 31-4428	55	Wuke 609	HYBRID 26-WK609
21	4430	INBRED LINE 32-4430	56	Xiongyu 587	HYBRID 27-XY587
22	4405	INBRED LINE 33-4405	57	Longdan 4	HYBRID 28-LD4H
23	4412	INBRED LINE 34-4412	58	Longyan 588	HYBRID 29-LY588
24	4411	INBRED LINE 35-4411	59	Nongda 4967	HYBRID 30-ND4967
25	4407	INBRED LINE 36-4407	60	Nongda 80	HYBRID 31-ND80
26	4246	INBRED LINE 37-4246	61	Zheng 58-PH4CV	HYBRID 35-Z58-PH4CV
27	4252	INBRED LINE 39-4252	62	Zhengdan 958	HYBRID 40-ZD958
28	4422	INBRED LINE 40-4422	63	Zheng 58-PH6WC	HYBRID 43-Z58-PH6WC
29	4410	INBRED LINE 41-4410	64	Jingke25	HYBRID 47-JK25
30	EH	INBRED LINE 44-EH	65	Jingdan 38	HYBRID 48-JD38
31	CC 15	INBRED LINE 45-CC15	66	MC 703	HYBRID 50-MC703
32	CC 14	INBRED LINE 47-CC14	67	Longdan 16	HYBRID 52-LD16
33	CC 13	INBRED LINE 48-CC13	68	Nongda 68	HYBRID 53-ND68
34	CC 16	INBRED LINE 49-CC16			
35	CC 12	INBRED LINE 50-CC12			

### 2.2 Acquisition of NIR spectral information

**Figure 1:**
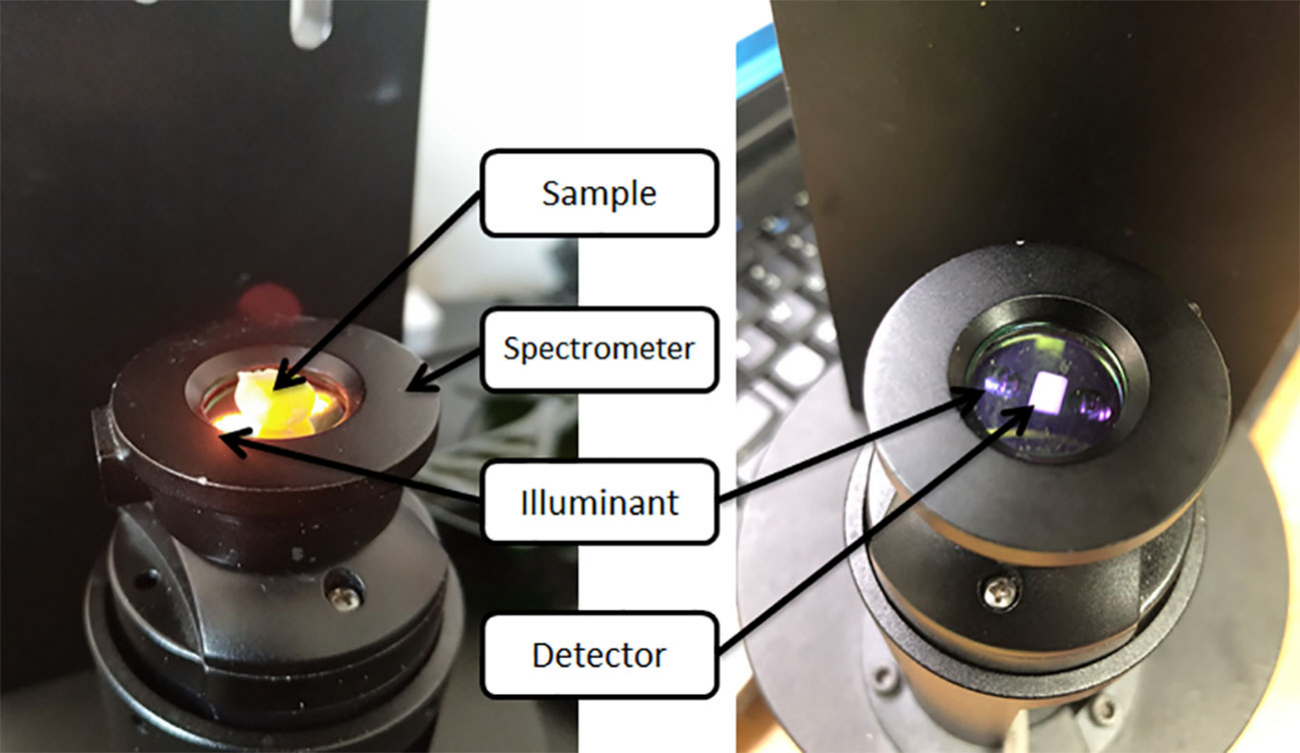
Instruments scheme of transmission mode.

Spectral data were acquired using a Micro NIR 1700ES near infrared spectrometer manufactured by JDSU (**Figure 1**). The spectral range was from 908.1 to 1677.2 nm, with the gap between neighbouring bands being 6.1944 nm, totalling 125 wavelength points (Pu et al., 2021). The spectroscopic equipment was warmed up for 45 min before use and a black and white reference correction was made with a BaSO_4_ correction whiteboard: the correction whiteboard was completely covered on the upper end of the spectrometer; the tungsten lamp was switched off and the dark correction was collected; the light was switched on and the white correction was collected. The spectral data of single seeds were collected in all experiments, and the embryonic part of the seeds was placed on one side of the detector, and five spectra were acquired for each sample, and the average value was taken as the raw spectral data of the samples. The details of the parameters of the spectral acquisition are as follows: the number of integrations is 200, the spectral acquisition time is 2 s (Agelet and Hurburgh, 2014).

### 2.3 NIR spectral data analysis

Spectral data were analyzed using the software The Unscrambler X version 10.5.1 (CAMO Analytics, Magnolia, TX, USA).

#### 2.3.1 Detection of spectral outliers

PCA is a commonly used technique for data dimensionality reduction. The original data is mapped by a linear transformation to a new coordinate system, where the coordinates are called principal components (Fabiyi et al., 2019). In the new coordinate system, the features according to the variance of the data from the largest to the smallest are located sequentially on different principal components. Thus, PCA can be used to detect outliers in spectral data. The acquired spectral data were utilized for PCA to detect spectral outliers as well as to explore common patterns between spectra.

#### 2.3.2 Spectral pre-processing

Savitzke-Golay Derivatives (SGD) and Standard Normal Variate (SNV) were chosen for spectral preprocessing to improve the signal-to-noise ratio of the spectra. SGD is based on the spectral smoothing results, and the derivative values of the spectral curves are calculated at different positions, highlighting spectral variations and features (Zhang and Mouazen, 2023). SNV is used to improve the comparability and interpretability of spectral data, making spectral variations between different samples more obvious, and is suitable for applications such as interpretation, classification and prediction of spectral data (Lanjewar et al., 2023).

#### 2.3.3 Establishment of authenticity identification model

In this experiment, we chose to use the SVM algorithm to build classification models. As a common supervised learning algorithm, the support vector machine is a binary classification model that maps the sample data into a high-dimensional space and then finds an optimal hyperplane in that space, which in turn separates samples of different categories. The choice of hyperplane is based on the principle of maximizing the separation of two samples of different categories (Devos et al., 2009). The Radial Basis Function (RBF) in this algorithm is a common kernel function for nonlinear classification, also known as Gaussian kernel function. It is calculated as follows:


$$\text{K}\left(\text{x},x^{\prime }\right)=\text{exp}\;(-\text{γ ||x}-x^{\prime }\;{\text{||}}^{2})$$

where x and x’ are the input vectors and γ is a hyperparameter controlling the decay rate of the function. ||x-x’||² denotes the square of the Euclidean distance. Specifically, the radial basis kernel function achieves classification of linearly indistinguishable problems by measuring the similarity between the samples and projecting the samples into a high-dimensional space centered on the support vectors (Wu et al., 2019).

The preprocessed spectral data were divided into modelling set and external test set according to 2:1.That is, 100 spectra of each material were randomly selected as the modelling set, and the remaining 50 spectra were used as the external test set, which were sequentially combined with the spectral data of other genotypes, and the optimal hyperplane needed to be found by grid searching during the selection of the SVM modelling process, and the model performance was cross-validated by the Leave-One-Out method (Kavdir et al., 2009). Then, the penalty factor C and kernel function parameter γ are determined by calculating the model training accuracy and cross-validation accuracy, and the optimal parameter combination is sought (Alves and Poppi, 2013). Finally, the performance of the recognition model is evaluated based on the accuracy of the external test set.

#### 2.3.4 Cluster analysis of spectral data

Based on the pre-processed spectral data, the Euclidean distance between the samples was calculated by using the dist function in R language and selecting the “Euclidean” method (Li et al., 2015). Then the Euclidean distance matrix was imported into Power Marker V3.25 software to obtain the Neighbor-Joining clustering results of the spectral data, and the clustering diagram was drawn with MEGA7 software.

### 2.4 DNA analysis of seed samples

The DNA of the samples was extracted by CTAB method (Wang et al., 2011). The 40 pairs of SSR primers used in this study are all primers published in the Chinese industry standard for maize variety identification. These primers have good polymorphism and are evenly distributed on the 10 chromosomes of maize. The specific primer names, sequences, and fragment lengths are referred to in published literature, and 40 pairs of cores SSR fluorescent primers were shown in **Table 2**. SSR-PCR system: The total volume of the reaction solution is 20 μL, including 10 μL of 2×Taq Plus Master Mix, 7.75 μL of ddH2O, 0.25 μL of primers, and 2 μL of DNA sample. PCR program: pre-denaturation at 95°C for 5 min; denaturation at 94°C for 40 s, annealing at 60°C for 35 s, extension at 72°C for 45 s, 35 cycles; extension at 72°C for 10 min; PCR products are stored at 4°C.

**Table 2:** Name, repeat unit, fragment range and chromosome location of 40 pairs of cores SSR primers.

No.	Primer name	Repeat unit	Fragment range(bp)	Chromosome location
P01	bnlg439w1	(TC)	321~369	1.03
P02	umc1335y5	(AG)	233~257	1.06
P03	umc2007y4	(TC)	233~300	2.04
P04	bnlg1940k7	(CT)	324~388	2.08
P05	umc2105k3	(AG)	280~350	3.00
P06	phi053k2	(GTAT)	333~363	3.05
P07	phi072k4	(TGTT)	408~432	4.01
P08	bhlg2291k4	(AG)	362~421	4.06
P09	umc1705w1	(CT)	254~349	5.03
P10	bnlg2305k4	(GA)	240~312	5.07
P11	bnlg161k8	(AG)	154~216	6.00
P12	bnlg1702k1	(CT)	260~347	6.05
P13	umc1545y2	(AAGA)	180~249	7.00
P14	umc1125y3	(CTCG)	149~175	7.04
P15	bnlg240k1	(GA)	220~239	8.06
P16	phi080k15	(GGAGA)	202~238	8.08
P17	phi065k9	(GTGAA)(GTGCA)	391~415	9.03
P18	umc1492y13	(GCA)	270~290	9.04
P19	umc1432y6	(TC)	211~259	10.02
P20	umc1506k12	(TTTG)	163~196	10.05
P21	umc1147y4	(CA)	149~172	1.07
P22	bnlg167y17	(CT)	173~255	1.10
P23	phi96100y1	(AGGT)	231~287	2.00
P24	umc1536k9	(GT)(TA)	216~238	2.07
P25	bnlg1520k1	(CT)(AC)(GA)(TA)	164~202	2.09
P26	umc1489y3	(GCG)	231~265	3.07
P27	bnlg490y4	(TA)	245~331	4.04
P28	umc1999y3	(TGC)	167~208	4.09
P29	umc2115k3	(GCCAT)	265~295	5.02
P30	umc1429y7	(AGC)	125~143	5.03
P31	bnlg249k2	(AG)	259~313	6.01
P32	phi299852y2	(CTG)	200~254	6.07
P33	umc2160k3	(AG)	198~244	7.01
P34	umc1936k4	(TG)	153~176	7.03
P35	bnlg2235y5	(TG)	174~198	8.02
P36	phi233376y1	(CCG)	180~222	8.09
P37	umc2084w2	(CTAG)	184~214	9.01
P38	umc1231k4	(GA)	239~283	9.05
P39	phi041y6	(CAGC)	296~334	10.00
P40	umc2163w3	(AG)	280~352	10.04

PCR product detection: A method of electrophoresis detection of 10-fold PCR products was used. 2 μL of 10-fold PCR mixed products and 10 μL of formamide containing 1% GS3730-500 molecular weight internal standard was added to individual wells of a 96-well electrophoresis plate. The above mixed samples were placed in a PCR instrument for denaturation at 95°C for 5min, stored at 4°C for 10 min, centrifuged at 2000 rpm for 30 s, and then electrophoresed on an ABI 3730XL DNA analyzer using fluorescence capillary electrophoresis (Wang et al., 2017). The electrophoresis time was 30 min and the raw data were collected by Data Collection software, and the data were genotyped and analyzed using SSR Analyzer (V1.2.4) fingerprint analyzer.

Genetic clustering was analyzed with reference to previous studies (Liu et al., 2021). SSR genotype data were analyzed using Power Marker V3.25 software, and genetic distances between samples were calculated based on Saitou and Nei's (1987) method to obtain the Neighbor-Joining clustering results of SSR markers, which were combined with MEGA7 software to draw the clustering diagram (Nei and Li, 1979).

## 3 Results and analysis

### 3.1 Genetic clustering of maize seed samples

**Figure 2:**
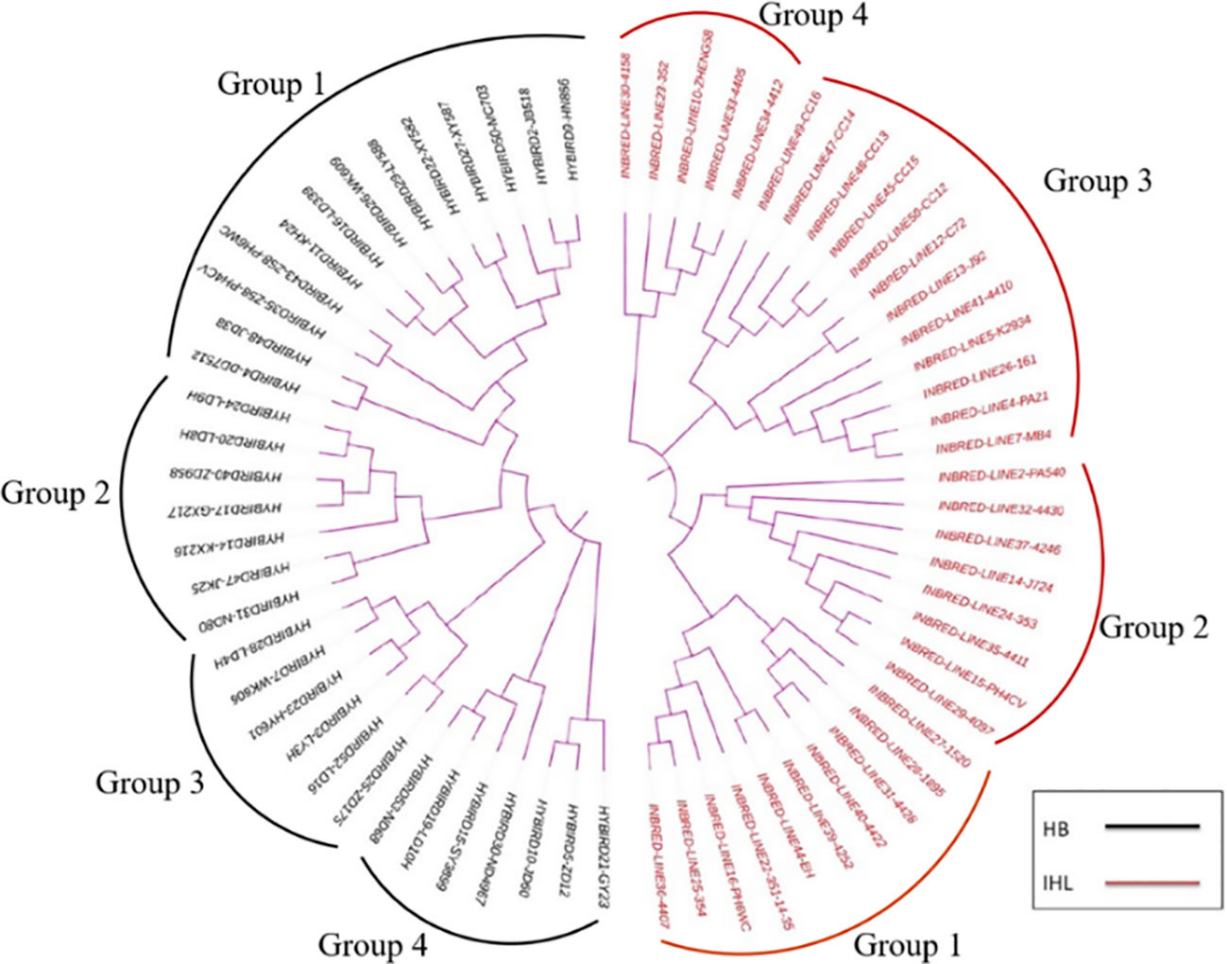
Genetic clustering plots between maize seed samples of different genotypes, with results based on genetic distances between samples, combined with the unweighted Pair-Group Method using Arithmetic averages. HB, hybrids; IHL, inbred lines.

The SSR genotype data was clustered by using the Unweighted Pair Group Method with Arithmetic Mean (UPGMA) method, and a clustering diagram was plotted using the MEGA7 software. Thirty-five inbreds and 33 hybrids were clustered and analyzed and the results are shown in **Figure 2**. 35 inbreds were clustered into four groups with a minimum genetic distance of 0.0250 and a maximum genetic distance of 0.9000. 33 hybrids were also clustered into four groups with a minimum genetic distance of 0.0500 and a maximum genetic distance of 0.8947.

### 3.2 NIR spectral characterization and spectral pre-processing

**Figure 3:**
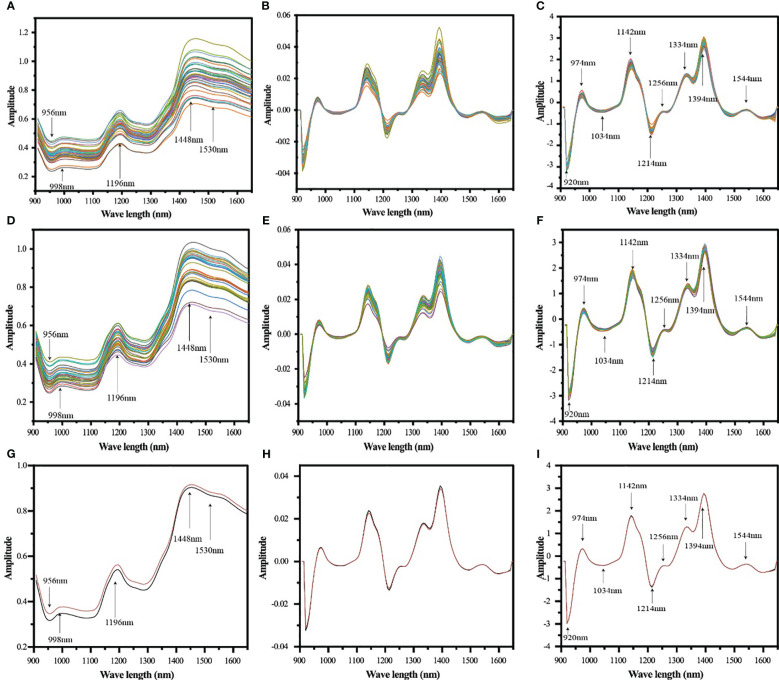
Average spectra of single grain samples of maize seeds of different genotypes acquired with Micro NIR 1700ES near infrared spectrometer (900~1700 nm) (**A**: inbred lines, **D**: hybrids, **G**: hybrids and inbred lines), spectra of SGD pretreatment (**B**: inbred lines, **E**: hybrids, **H**: hybrids and inbred lines), and SGD+SNV pretreatment spectra (**C**: inbred lines, **F**: hybrids, **I**: hybrids and inbred lines), with different colored lines reflecting the average spectra of maize seed samples of different genotypes, and the red and black lines in **(G–I)** represent the average spectra of inbred lines and hybrids, respectively.

**Figure 4:**
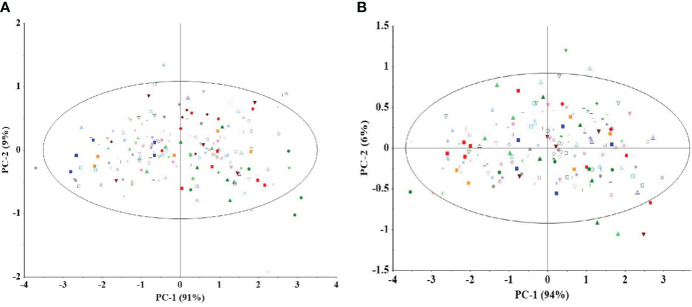
Score plots using the first two components of all NIR spectra (mean centers). Principal component analysis was performed on the spectral data of inbred lines **(A)** and hybrids **(B)**. In the figure, each point of the same color and shape represents the distribution of different inbred lines **(A)** or hybrid **(B)** (5 spectral data were randomly selected for each genotype sample) in the first two principal component spaces. PCA analysis of inbred lines and hybrids cannot effectively distinguish different genotypes based on spectra.

Single kernel detection, combined with diffuse reflectance, was used to collect spectral information from the embryo surface position of the maize samples, and the spectral features are detailed in **Figure 3**. Subsequently, the spectral information was subjected to principal component analysis, using mean-centered data. The results indicate that no outliers were found in any of the datasets. As shown in **Figure 4**, the graph demonstrates the first 2 PC score plots. From the figure, it is easy to find that the different genotypes of maize seeds show the trend of clustering, which suggests that the NIR spectral information contains information related to the genotypes of the samples. However, PCA analysis of inbred lines and hybrids cannot effectively distinguish different genotypes of samples based on spectra.

The spectral data were preprocessed using SGD and SNV. It can be seen from **Figure 3** that the spectral peak changes of the pretreated near-infrared spectra are clearer, especially the baseline shift has been significantly corrected.

### 3.3 NIR spectral clustering of maize seeds

**Figure 5:**
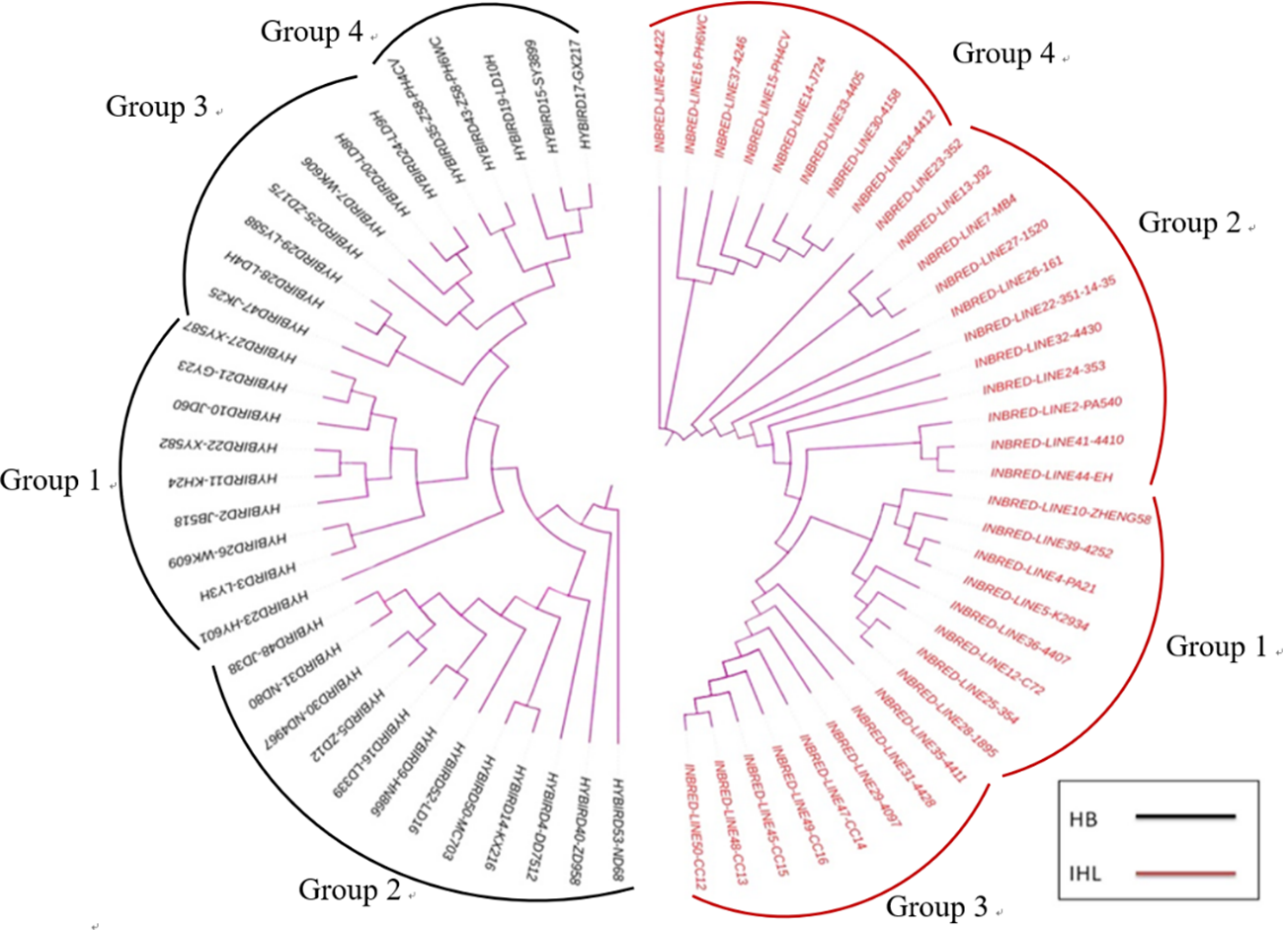
Spectral clustering plots between maize seed samples of different genotypes. results were based on the relative spectral distances between samples combined with the unweighted Pair-Group Method using Arithmetic averages. HB: hybrids; IHL: inbred lines.

Spectral clustering analysis was carried out using the preprocessed spectral data. In **Figure 5**, 35 inbred samples were clustered into four groups, while 33 hybrid samples were also clustered into four groups. Subsequent comparison of the genetic clustering results with the spectral clustering results revealed that among the inbreds, 33 samples, or 94.3 per cent of the total, had the same clustering. Among the hybrids, 31 samples had the same clustering, accounting for 93.9% of the total. It can be seen that to a certain extent the NIR spectra can reflect the genetic relationship between maize seeds of different genotypes.

### 3.4 Correlation analysis of relative spectral distances and genetic distances

The Mantel function was used to correlate the two dissimilarity matrices, relative spectral distance and genetic distance (Wynn et al., 2016). This was done specifically with the mantel function in the R language vegan package. Permutation was done with free permutation and Permutation defaulted to 999. The results of the Mantel test, as shown in **Table 3**, showed that the two matrices of relative spectral and genetic distances were significantly and positively correlated among the 35 inbred lines (Mantel test; r=0.177, *p*<0.05), and 33 hybrids showed the similar situation (Mantel test; r=0.238, *p*<0.05).

**Table 3:** Results of Mantel test for genetic distance and relative spectral distance among maize seed samples of different genotypes.

Sample name	Upper quantiles of permutations (null model) (90%;95%; 97.5%;99%)	Permutation	Number of permutations	Mantel statistic r	Significance
IBL	0.0799;0.1008; 0.1225;0.1425	Free	999	0.8907	0.035
HB	0.110;0.135; 0.159;0.200	Free	999	0.8614	0.045

HB, Hybrid; IBL, Inbred Line.

### 3.5 Establishment of authenticity identification model

**Figure 6:**
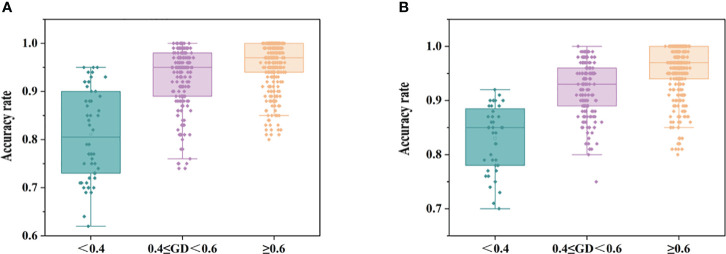
Acc statistics of authenticity discrimination models for maize seed samples of different genotypes (**A**: inbred lines, **B**: hybrids).

**Figure 6** shows the discriminative performance of the model. The average accuracy of the models was 93.6%, and the accuracy of all models was distributed between 62% and 100% for 35 maize inbred lines with 595 discriminant models. A total of 528 discriminatory models were established for 33 maize hybrids, and the average accuracy of the models was 93.7%, with the accuracy of all models ranging from 76% to 100%.

### 3.6 Relationship between genetic distance and model discriminatory performance

**Figure 7:**
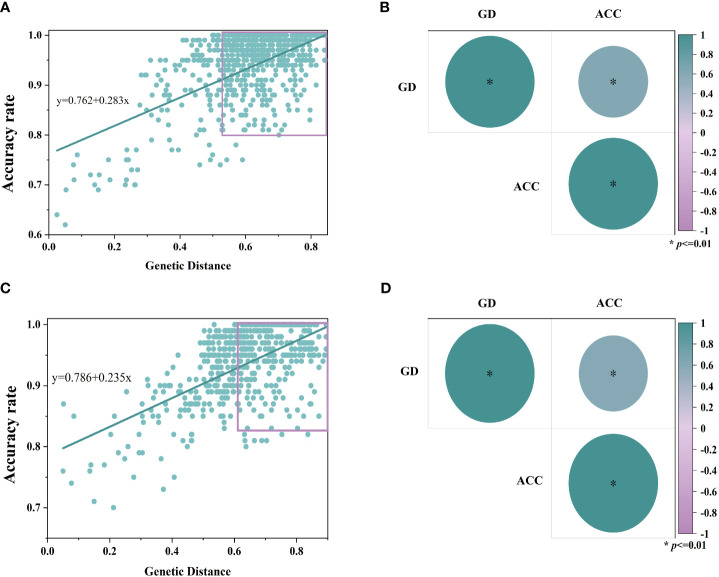
Results of simple linear correlation analysis of genetic distance between samples and accuracy rate of the model. Where **(A, C)** are linear regression fitting curves for inbred lines and hybrids, respectively, and **(B, D)** are bivariate Pearson test results for inbred lines and hybrids (*p*<0.01), respectively. (GD, genetic distance; ACC, accuracy rate).

In order to resolve the effect of genetic distance on model accuracy, simple linear correlation analyses were performed on the above two sets of data, and the bivariate Pearson’s test showed positive correlation between genetic distance and model accuracy in the inbred samples (r=0.611, *p*<0.01), and the hybrid samples as well (r=0.6158, *p*<0.01), as shown in **Figure 7**.

The accuracy of the model increases gradually with the genetic distance in **Figure 7**. When the genetic distance is more than 0.4, the average accuracies of the inbred and hybrid discrimination models are 94.9% and 94.6%, respectively; when the genetic distance is more than 0.6, the average accuracies of the models increase to 95.9% and 95.8%, respectively. This reveals that there is positive correlation between the performance of the NIR spectral discrimination model and the genetic distance of the samples, with the nearer the genetic distance, the lower the accuracy of the model.

## 4 Discussion

Firstly, in this work, the spectral data of single kernel of maize samples were acquired by portable near-infrared spectrometer (900~1700nm), combined with SGD and SNV data preprocessing algorithms to establish SVM classification model, and the average accuracy of inbred and hybrid samples were 93.6% and 93.7%, respectively, which proved the feasibility of this technology in the field of seed authenticity detection (McVey et al., 2021). NIR spectroscopy has medium energy and strong transmission ability, and mainly responds to the vibrational information of atomic groups such as C-H, N-H, O-H, and so on (Bec et al., 2021). While the nutritional qualities such as crude protein, crude fat, crude starch and lysine content ranged from 8.18% to 12.64%, 3.41% to 4.77%, 71.44% to 77.67% and 0.238% to 0.42% with coefficients of variation of 0.10%, 0.08%, 0.02% and 0.12% among different samples, respectively. This variation is reflected in the NIR spectral information, which in turn leads to excellent model performance.

**Figure 8:**
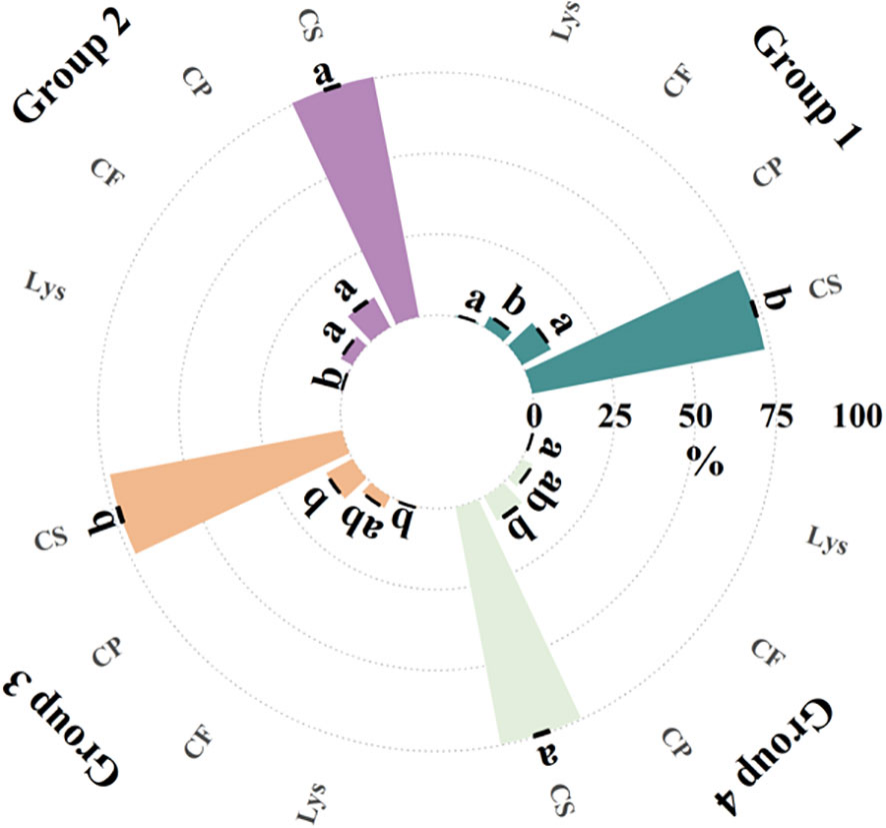
Biomolecule content of hybrid samples in different spectral clustering groupings. Different letters in the figure indicate significant differences in the same indicator at the *p*<0.05 level. (CP, crude protein; CS, crude starch; CF, crude fat; Lys, lysine).

Secondly, the clustering analysis of the hybrid samples indicates that there are differences in the content of biological macromolecules among different groups. These differences may be caused by the genetic characteristics of the samples, because the influence of genetic characteristics on seed chemical components accounts for about 18% of the variation (Ferreira et al., 2014). We therefore analyzed the biomolecule content of the hybrids and found differences in crude protein, crude starch, crude fat and lysine content between the four groups of samples from the spectral clustering results, as shown in **Figure 8**. Previous studies on the identification of 25 maize materials through chemical composition and SSR markers showed that Mantel test results indicated a significant positive correlation between seed chemical composition and SSR molecular marker genetic distance (Sofy et al., 2020).

Thirdly, this study found that as the genetic distance increased, the relative spectral distance also increased, along with the accuracy of the model. The researchers found some kind of possible connection between the genetic and relative spectral distances between the samples and the performance of the model, and suggested that the discriminatory performance of the model improved as the genetic distance increased (Liu et al., 2015). However, the above survey involved only five maize inbred materials, while 35 inbreds and 33 hybrids were selected for comparative validation in this work; moreover, portable spectrometers as well as single grain detection are more suitable for online application scenarios of massive quantities and meet the actual needs of production (Pu et al., 2021).

Finally, 5.72% and 6.06% of the two clustering outcomes in the inbreds and hybrids, respectively, differed in this study, which may be mainly due to the effect of the performance of the spectroscopic instruments themselves (Agelet and Hurburgh, 2014). Some of the characteristic bands reflecting the inclusions of the samples may be missing in the wavelength range of 900~1700 nm, i.e., in the long-wave NIR spectral region of 1700~2500 nm, 1923 nm and 2009 nm carry information on the combined frequency of the O-H group in water and starch, whereas 2125 nm and 2173 nm carry information on the diploid frequency of the N-H group in proteins (Ozaki et al., 2007).

This work assessed the applicability of portable near-infrared spectroscopy to identify the authenticity of single maize seeds. The seed samples selected covered both inbred lines and hybrids, which ensures that the technique can be used as a rapid and non-destructive tool for multiple scenarios of maize seed detection in the field, in warehouses, and online by a diverse group of people such as breeders, producers, and inspectors.

## 5 Conclusion

The application of NIR spectroscopy to detect the authenticity of crop seeds is not only affected by instrument parameters, preprocessing algorithms, modelling approaches, etc., but the seed genetic distance, as an indicator reflecting the genetic background among the samples, also affects the discrimination performance of the identification model. In this work, a portable near-infrared spectrometer was applied to systematically study for the first time the relationship between genetic distance, relative spectral distance and model performance. In addition, the genetic clustering results performed consistently with the spectral clustering results. NIR spectroscopy can well reflect the genetic relationship between corn seeds. The calculation of relative spectral distance is faster than molecular marker detection methods, and it has lower requirements for operators than establishing classification models. Therefore, in the future, it can be achieved to quickly identify the genetic background of single seeds using spectral information of the samples, effectively shortening the time limit of crop breeding. This discovery brings good news to breeders and seed producers. In summary, NIR spectroscopy is of great value to modern crop breeding, variety identity, and purity sorting as an assistant means of genetic breeding.

## Data availability statement

The original contributions presented in the study are included in the article/supplementary material. Further inquiries can be directed to the corresponding author.

## Author contributions

YQY: Data curation, Formal analysis, Software, Writing – original draft, Writing – review & editing. RH: Writing – original draft. DZ: Methodology, Writing – review & editing. BS: Formal analysis, Methodology, Writing – original draft. YLY: Funding acquisition, Writing – original draft. DK: Conceptualization, Funding acquisition, Project administration, Software, Supervision, Writing – review & editing.
